# Oxidative Stress Induced Mitochondrial Failure and Vascular Hypoperfusion as a Key Initiator for the Development of Alzheimer Disease 

**DOI:** 10.3390/ph3010158

**Published:** 2010-01-19

**Authors:** Gjumrakch Aliev, Hector H. Palacios, Eldar Gasimov, Mark E. Obrenovich, Ludis Morales, Jerzy Leszek, Valentin Bragin, Arturo Solís Herrera, Dmitry Gokhman

**Affiliations:** 1 School of Health Science and Healthcare Administration, University of Atlanta, 6685 Peachtree Industrial Blvd., Atlanta, Georgia, 30360, USA; 2 Department of Nutrition and Biochemistry, Faculty of Sciences, Javeriana University, Bogotà D.C., Colombia; 3 Stress Relief and Memory Training Center, Brooklyn, New York, NY 11235, USA; 4 Department of Biology, College of Sciences, University of Texas at San Antonio, One UTSA Circle, San Antonio, TX 78249–1664, USA; 5 Department of Cytology, Histology and Embryology, Azerbaijan Medical University, 25 Street Bakhikhanov, Baku AZ10 25, Azerbaijan; 6 Department of Pathology, School of Medicine, Case Western Reserve University, WRB 5301, Cleveland, Ohio, 44106, USA; 7 Department of Psychiatry, Wroclaw Medical University, 25 St. Kraszewskiego, Wroclaw, 50–229, Poland; 8 Dirección de Investigación y desarrollo, Centro de Estudios de la Fotosíntesis Humana, S.C. López Velarde 108 y 109, Centro, Aguascalientes, Aguascalientes, 20000, México; 9 Department of Mathematics, College of Sciences, University of Texas at San Antonio, One UTSA Circle, San Antonio, TX 78249, USA

**Keywords:** oxidative stress, Alzheimer disease, antioxidants, hypometabolism, mitochondria, metabolism, neurodegeneration

## Abstract

Mitochondrial dysfunction may be a principal underlying event in aging, including age-associated brain degeneration. Mitochondria provide energy for basic metabolic processes. Their decay with age impairs cellular metabolism and leads to a decline of cellular function. Alzheimer disease (AD) and cerebrovascular accidents (CVAs) are two leading causes of age-related dementia. Increasing evidence strongly supports the theory that oxidative stress, largely due to reactive oxygen species (ROS), induces mitochondrial damage, which arises from chronic hypoperfusion and is primarily responsible for the pathogenesis that underlies both disease processes. Mitochondrial membrane potential, respiratory control ratios and cellular oxygen consumption decline with age and correlate with increased oxidant production. The sustained hypoperfusion and oxidative stress in brain tissues can stimulate the expression of nitric oxide synthases (NOSs) and brain endothelium probably increase the accumulation of oxidative stress products, which therefore contributes to blood brain barrier (BBB) breakdown and brain parenchymal cell damage. Determining the mechanisms behind these imbalances may provide crucial information in the development of new, more effective therapies for stroke and AD patients in the near future.

## 1. Introduction

Alzheimer disease (AD) and cerebrovascular accidents (CVAs) are two leading causes of age-related dementia. Increasing evidence supports the notion that chronic hypoperfusion is primarily responsible for the pathogenesis that underlies both disease processes. In this regard, hypoperfusion appears to induce oxidative stress, which is largely due to the formation of reactive oxygen species. Oxidative imbalance is also associated with other age-related degenerative disorders such as atherosclerosis, ischemia/reperfusion, and rheumatic disorders. 

We have found that a chronic injury stimulus induces the hypoperfusion seen in the microcirculation of vulnerable brain regions. This leads to energy failure, which is manifested by damaged mitochondrial ultrastructure, the formation of a large number of non-mature or “young” electron dense “hypoxic” mitochondria and by the overproduction of mitochondrial DNA (mtDNA) deletions. Moreover, these mitochondrial abnormalities coexist with increased redox metal activity, lipid peroxidation and RNA oxidation. This oxidative stress occurs within various cellular compartments, in various parenchymal cells in the brain and most notably in the vascular endothelium, and in mitochondria found therein, which is associated with atherosclerotic damage. Further, the associated pathology is accompanied by neuronal and glial damage, known to be a part of the development of AD pathology. In addition, vascular wall cell pathology in the AD brain correlates linearly with the degree of neuronal and glial cell damage. Mitochondrial lesions in all of these cellular compartments show the same pattern, namely DNA deletions, the overexpression of oxidative stress and appears strongly to be the central target for brain damage in AD, due to high energy demand and susceptibility to oxidation. The result is manifested as energy failure and results in cognitive impairment and memory decline. In this review we outline recent evidence, as well as our own experimental data, indicating that chronic injury–stimulus induces hypoperfusion in the microcirculation of vulnerable brain regions, which leads to energy failure. 

## 2. Vascular Changes and Their Influence in the Pathology Seen in AD

Recent findings demonstrate that there is a similarity between the ultrastructural features of both vascular lesions and mitochondria in brain vascular wall cells from human AD brain biopsies, human short postmortem brain tissues, yeast artificial chromosome (YAC R140) and C57B6/SJL Tg+ mice overexpressing Aß precursor protein (PP) [1,2]. Performing in situ hybridization using mtDNA probes for human wild type, 5kb deleted and mouse mtDNA, and immunocytochemistry using antibodies against APP, 8-hydroxyl-2’-guanosine (8OHG) and cytochrome c oxidase subunit 1 (COX) provide congruent ultrastructural localization [1,2]. A higher degree of amyloid deposition in the vascular walls of the human AD, YAC and C57B6/SJL Tg (+) mice exists compared to age-matched controls [1]. Severely damaged vessels exhibit immunopositive staining for APP. More mitochondrial abnormalities are present in human AD, YAC and C57B6/SJL Tg (+) mouse microvessels where lesions occur [1,2]. Undamaged regions of human AD tissues, YAC and C57B6/SJL Tg (+) mouse tissues and in age-matched control subjects lack these features, while damaged vessels manifest cells possessing clusters of wild and deleted mtDNA–containing positive probes [1,2]. Our observations demonstrate that vascular wall cells, especially their mitochondria, appear to be central targets for oxidative stress-induced damage before the development of AD pathology [1,2]. On the other hand, the positive correlation between AD and cholesterol levels suggests that antioxidant therapy and cholesterol–lowering drugs could delay the occurrence of AD [1]. However, despite their frequencies, the pathophysiological and morphological changes in brain microcirculation that accompany AD remain poorly understood, and the specific factors controlling vascular tone in AD remain unknown [2]. 

## 3. Features That Influence the Development and Prognosis of AD during the Interactions between Cerebrovascular Diseases and Dementia

The role of tobacco smoking in the pathogenesis of AD is still unclear and controversial. ROS are generated at sites of injury and/or inflammation. The vascular endothelium, which regulates the passage of macromolecules and circulating cells from blood to tissue, is a major target of oxidant stress and plays a critical role in the pathophysiology of several vascular diseases. In addition, the vascular endothelium, neurons, and glia can synthesize, store, and release ROS and vasoactive substances in response to certain stimuli, especially to chronic hypoxia/hypoperfusion. Their contribution to the pathophysiology of stroke, cerebrovascular disease and AD is extremely important. Moreover, the role of hypoperfusion as a key factor for vascular lesions that causes oxidative stress, appears to be widely accepted as an initiator of AD [1,2]. This idea is based on a positive correlation between AD and cardiovascular diseases [1,3,4,5,6].

Specifically, accumulated oxidative stress increases vascular endothelial permeability and promotes leukocyte adhesions, which is coupled with alterations in endothelial signal transduction and redox–regulated transcription factors [for the references and review see: [1,3,4]]. We hypothesize that the cellular and molecular mechanisms by which Hypoperfusion-induced ROS accumulation results in the development of AD is through impairing endothelial barrier function, promoting leukocyte adhesion and altering normal vascular function. The sustained hypoperfusion and oxidative stress of brain tissues also could stimulate the secondary overexpression of iNOS and nNOS and endothelin–1 (ET-1) in brain cells [7]. It is likely that the increased accumulation of oxidative stress products probably contributes to damage to brain parenchymal cells and the decompensation of the blood brain barrier (BBB), which normally prevents permeability of large molecules from passing to the cerebrospinal fluid. Therefore, determining the mechanisms behind these disturbances in experimental animals may provide crucial information in the development of new, more effective therapies for the treatment of cerebrovascular and neurodegenerative diseases, including AD.

Many common underlying risk factors play key roles in the development of cardiovascular, cerebrovascular and neurodegenerative diseases [4,8,9,10]. Cigarette smoking causes chronic hypoxic conditions and the formation of a large amount of free oxygen radicals that appear to be key factors in the development of these diseases. Latest evidence indicates that continuous exposure to free oxygen radicals induce cellular damage and decreases antioxidant defenses [11].

Several recent studies indicate that cigarette smoking is a cofactor in the initiation of AD via its effect on the vasculature, as previously discussed. Nicotine via nicotinic receptor activation may counter these effects in part. Vascular insufficiency/hypoperfusion has been considered as a pathogenetic factor in the development of AD, and the positive relationship between cerebrovascular diseases such as stroke and especially cerebrovascular atherosclerosis indicates the latter may also be linked to the pathogenesis of AD [4].

## 4. The Influence of Oxidative Stress on the Function of Brain Microvessels in AD 

ROS can function as signaling intermediates at low levels and regulate fundamental cell activities including growth and adaptive responses [11]. However, at higher concentrations, ROS can cause cell injury and death. Vascular endothelium modulates the passage of macromolecules and circulating cells from blood to tissue and is a major target of oxidant stress [12]. Specifically, oxidative stress increases vascular endothelial permeability and promotes leukocyte adhesions, which are coupled with alterations in endothelial signal transduction and redox-regulated transcription factors [12]. Based on these recent findings, we hypothesize that impairing endothelial barrier function and promoting leukocyte adhesion also induce alterations in normal vascular endothelial cell (EC) function, resulting in AD progression [12].

Compared to other organs or tissues, the brain is more vulnerable to ROS-induced damage due to its high rate of oxygen consumption, high polyunsaturated lipid content, and relative paucity of classic antioxidant enzymes [13]. The AD brain contains increased regional levels of oxidative stress indicators [14,15,16,17,18,19,20]. Studies demonstrate a decline in polyunsaturated fatty acids (PUFA) [21,22,23], increased levels of lipid peroxidation markers [19,21], as well as protein oxidation [24,25], DNA oxidation [26,27,28] and RNA oxidation [1,29,30,31] during AD. Additionally, the presence of advanced glycation end products (AGE), glycoxidative end products such as N–ε–carboxy–methyl–lysine and lipid peroxidation adducts are present in both neurofibrillary tangles (NFT) and senile plaques (SP) in AD [1,15,18,19,24,25,29,30,31,32,33,34] as well as in post–ischemic tissues [35,36,37,38,39].

Vascular aging correlates with both structural and functional changes that can take place at the level of the endothelium, vascular smooth muscle cells (vSMC) and the extracellular matrix of blood vessels. In the endothelium, reduced vasodilatation in response to agonists occurs in large conduit arteries, as well as in resistance arteries as a result of aging [40]. Furthermore, enhanced oxidative stress by hypoperfusion contributes significantly to the deleterious effects of aging on the endothelium by means of NO breakdown due to ROS. The relative contribution of the above phenomenon to age-related endothelial dysfunction is highly dependent on the species and the type of vascular bed involved [9,40,41,42].

Cortical, subcortical, meningeal gray matter and blood vessels (congophilic angiopathy) all contain Aβ deposits and are prominent features of AD [5,6,9,40,41,42]. *In vitro* experimental evidence shows that these Aβ deposits induce cerebrovascular dysfunction in the rat brain [43], and that the beta amyloid (Aβ) peptide produces endothelial dysfunction in cerebral microvessels via ROS. This occurs when the ROS superoxide–scavenging enzyme, superoxide dismutase, prevents acetylcholine–induced endothelium–dependent vasodilation [43]. In addition, accumulating evidence supports the idea that the Aß peptide is responsible for the cerebrovascular effects of the upstream molecule beta amyloid precursor protein (AßPP) and its overexpression [44,45].

A study by Iadecola and coworkers shows how transgenic (Tg) mice overexpressing AßPP have a profound and selective impairment in endothelium–dependent regulation of neocortical microcirculation [39]. Moreover, peptides derived from AßPP processing may contribute to the alterations in cerebral blood flow (CBF) and neuronal dysfunction during AD [44]. The study confirmed that Aß1-40 did not influence the increasing CBF produced by the endothelium–independent vasodilators [40]. In contrast, Aß1-42 did not reduce resting CBF or the increasing CBF produced by endothelium–dependent vasodilators. The superoxide scavengers, SOD and MnTBAP, reversed the cerebrovascular effects of Aß1-40 [40,41]. These data strongly suggests that soluble amyloid beta protein (Aß1-40), but not amyloid aggregate (Aß1-42), produces the cerebrovascular alterations seen in transgenic AßPP mouse, and thus, Aß1-40 could play a role in the cerebrovascular alterations observed in AD [6,45]. This study supports recent evidence that microvessels isolated from the AD brain kill neurons *in vitro* [46].

The growing body of evidence suggests that AD shares many common underlying etiologies with other neurodegenerative disorders. For example, multiple sclerosis (MS) is a relatively common disease with no cure. It is the leading cause of neurological disability in young adults, affecting over two million people worldwide (reviewed in reference [47]). Traditionally, MS has been considered a chronic, inflammatory disorder of the central white matter in which ensuing demyelination results in physical disability. Recently, MS has become increasingly viewed as a neurodegenerative disorder in which axonal injury, neuronal loss, and atrophy of the central nervous system leads to permanent neurological and clinical disability. The latest developments on MS research, includes etiology, pathology, genetic association, EAE animal models, mechanisms of neuronal injury and axonal transport, and therapeutics [47]. Moreover, the mechanisms of mitochondrial dysfunction that are involved in MS, including mitochondrial DNA defects and mitochondrial structural/functional changes that accompanies this devastative disease have been able to open new and much more effective treatment strategies [47]. However, despite all the research on the effects of unknown etiology as well as Aß, the source of the potential ROS *in vivo* and its link to mitochondrial dependent hypoperfusion is not completely understood.

## 5. The Role of Mitochondrial Abnormalities during the Development of AD 

In aerobic cells 90–95% of the total amount of adenosine triphosphate (ATP) production requires oxygen. The synthesis of ATP via the mitochondrial respiratory chain is the result of electron transport across the electron transport chain coupled to oxidative phosphorylation [48]. The main radical produced by mitochondria is superoxide anion. Intramitochondrial antioxidant systems scavenge this radical to avoid oxidative damage, which can lead to impaired ATP production [49,50,51,52]. During aging and some neurodegenerative diseases, including AD, damaged mitochondria are unable to maintain the energy demands of the cell [53,54]. This can lead to an increased production of free radicals, inducing the interruption of oxidative phosphorylation, and resulting in decreased levels of ATP [51]. Both processes, defective ATP production and increased oxygen radicals, may induce mitochondria–dependent cell death [51].

Animal studies using mitochondrial toxins provide the association between neurodegeneration with mitochondrial dysfunction and oxidative damage [2,51]. These consequences are implicated in the pathogenesis of human as well as animal models of neurodegenerative diseases [55,56,57,58] and in particular AD [1,49,50,53,59,60,61,62]. After long–term ischemia/reperfusion the mitochondria ultra-structure disintegrates *in vivo* and *in vitro* [9,38,39]. Apoptosis of degenerating neurons occurs in association with the accumulation of perikaryal mitochondria and oxidative damage to the nucleus [63]. This same pattern of mitochondrial lesions is observed in human AD brain biopsy samples [53,59]. The reduced expression of both mtDNA and nuclear DNA encoded genes is consistent with a physiological down–regulation of the mitochondria respiratory chain in response to declining neuronal activity [49,50,51,52,53,54,55,56,57,58,61,62,64]. However, the role of somatic cells and mtDNA mutations in the pathogenesis of mitochondria failure during AD is still controversial [50,58,61,62]. 

The deleted mtDNA increases at least 3-fold in AD cases as compared to controls in humans [53]. Moreover, mtDNA isolated from the brains of AD patients includes oxidative modifications containing 8-hydroxy-2’-deoxyguanosine (8OHdG) [26,27,28]. Studies using in situ markers for 8OHdG and 8OHG showed that RNA oxidation is a prominent feature of damaged neurons in AD [29,30,31]. Quantitative analysis revealed a strong positive correlation between mtDNA deletions and 8OHG (r = 0.934) [53]. However, given that mtDNA (even DNA containing the 5kb deletion) is spared relative to 8OHG, we suspect that mitochondrial abnormalities correlate, but do not directly produce ROS. Therefore, it is important to recognize that 8OHG is formed by the direct attack of OHo. These OHo radicals have only a 2 nm sphere of diffusion and thus are unable to diffuse through the mitochondrial membrane [53]. 

More recently polarographic studies by Cormier and coworkers describe the effects of nicotine on respiratory chain in rat brain mitochondria [65]. The measurements of oxygen consumption show significant concentration–dependent inhibition by nicotine. Nicotine binds to complex I of the respiratory chain, inhibits NADH-Ubiquinone reductase activity and competes with NADH for complex I [65]. Furthermore, nornicotine, but not cotinine, the main nicotine metabolite, inhibits mitochondrial respiration. Complex I generates superoxide anion, nicotine, and was able to inhibit this ROS generation [65]. This may explain a part of the beneficial and protective effects of nicotine in a few neurodegenerative diseases, as suggested by many epidemiological studies [65]. However, future studies should focus on elucidating the effect of nicotine on the mitochondria functions as well as DNA overexpression and/or deletion during the development of neurodegenerative disorders including AD. The exact cellular mechanisms behind vascular lesions and their relation to oxidative stress markers identified by RNA oxidation, lipid peroxidation, or mtDNA deletion remain unknown [66]. Future studies comparing the spectrum of oxidative stress-induced damage during reperfusion injury or, more importantly, during hypoxia/hypoperfusion, with AD damage are warranted [67].

## 6. Co–Factors for Oxidative Stress-Induced Cerebrovascular Lesions

### 6.1. Hypoperfusion-Induced Oxidative Stress as a Key Factor for the Development of AD

Hypoperfusion-induced oxidative stress in vascular abnormalities coincides with the pathogenesis of AD [62]. Several studies conclude that chronic cerebral hypoperfusion in AD is secondary to oxygen reduction [10,68,69,70,71]. However, recent evidence reveals that a greater fraction of oxygen is removed from the vasculature in AD patients as compared to non–AD controls [72]. This suggests that low vascular blood flow is a prominent feature of the brain during chronic hypoxia/hypoperfusion and may be a main initiating factor during the development of AD [4,73,74]. An impairment of energy metabolism characterizes the AD brain [60]. Positron emission tomography (PET) reveals a decline in the cerebral metabolic rate of the parietal and temporal lobes during AD [25,75]. These metabolic defects are present before AD symptoms develop in ApoE ε4 homozygote patients [25]. De la Torre [73,76] proposes that advanced aging with a comorbid condition, such as a vascular risk factor that further decreases cerebral perfusion, promotes a critically attained threshold of cerebral hypoperfusion (CATCH). With time, CATCH induces brain capillary degeneration and suboptimal delivery of energy substrates to neuronal tissue [73,76]. Because glucose is the main fuel of brain cells, its impaired delivery, together with a deficient delivery of oxygen, compromise neuronal stability because the substrates for aerobic glycolysis fail to meet brain tissue demand. The outcome of CATCH is a metabolic cascade that involves, among other things, mitochondrial dysfunction, oxidative stress, decreased ATP production and increased calcium entry, abnormal protein synthesis, cell ionic pump deficiency, signal transduction defects, and neurotransmission failure. These events contribute to the characteristic progressive cognitive decline of patients with AD, as well as regional anatomic pathology, consisting of synaptic loss, SP, NFT, tissue atrophy, and neurodegeneration. CATCH characterizes the clinical heterogenic pattern of AD and provides compelling evidence that a multitude of etiopathophysiologic vascular risk factors, in the presence of advanced aging, can lead to AD [2,3,4,10,73,76,77,78,79]. Therefore, we hypothesize that taken together with vascular EC and SMC atrophy, hypoperfusion is a key factor in the development of AD.

### 6.2. Cerebrovascular Lesions Observed During Ischemia/Reperfusion Induced Oxidative Stress

The risk for Alzheimer dementia and stroke are known to increase at comparable rates with age. Recent advances suggest that vascular risk factors linked to cerebrovascular disease and stroke in the elderly significantly increase this risk [6]. Although some vascular lesions such as cerebral amyloid angiopathy, endothelial degeneration, and periventricular white matter lesions are evident in most AD cases, one third will exhibit cerebral infarction. Longitudinal clinical studies suggest that stroke and AD occur in tandem more often than randomly [80]. Strokes often occur in patients with AD and have been linked to the pathogenesis of dementia [6]. Nevertheless, the nature of this relationship remains unexplored. Cerebral ischemia is a possible causal factor for AD. Irrespective of the ultimate pathogenic mechanism, these findings suggest that managing vascular disease is important in the treatment and prevention of AD [8,10] or mixed dementia [6].

Chronic hypoxia can alter cerebral microvessels ultrastructure, but this effect is heterogeneous and in some cases capillaries can respond to hypoxia independently of the arteriole [81]. Exposure to three weeks of hypobaric hypoxia results in increased capillary density in rat models [82]. Capillary segment elongation plays a role in this increase in the deeper layers of the cerebral cortex [82]. Therefore, prolonged hypoxia results in structural and functional adaptive responses that improve tissue oxygen delivery [83]. Mitochondria of brain capillary EC maintain normal density in hypoxia, but the number of mitochondria in the surrounding neuropil decreases significantly about 30% [84]. Moreover, exposure to hypobaric hypoxia yields an increase in basic fibroblastic growth factor (bFGF) mRNA in brain tissue [85]. During moderate hypobaric hypoxia, increased brain vasculature is associated with increased density of the brain capillary glucose transporter (Glut-1). However, this change is reversible and dependent on hypoxia exposure time [86]. This same pattern has been observed in the microvascular system of the human AD brain [1,87,88,89]. Based on these findings, the relationship between oxidative stress markers and extracellular matrix binding ligands in the hypoxic brain during stroke and AD deserves further investigation. In addition, the injury induced by reperfusion after chronic hypoxia is important to note because the oxidative products that accumulate during hypoxia induce more tissue and cellular damage than the hypoxia itself. 

Ischemia/reperfusion is a systemic process affecting the whole organ or tissue. Different types of blood cells may contribute to the pathogenesis of ischemia/reperfusion, including platelets, monocytes, neutrophils and others [35]. According to Bednar and coworkers [90] neutrophils might be important contributors to ischemia–induced brain injury whereas the role of platelets is more nebulous. In fact, systemic depletion of neutrophils reduced the volume of cerebral infarct after transient middle cerebral artery occlusion in the rat [91]**.** EC affected by ischemia during the early stages is completely reversible and dependent upon reperfusion. Eventually, however, injured tissue passes a *“point of no–return”* and the damage becomes irreversible [92]. Initially, cells strive to increase their surface area for gas and nutrient exchange by expressing cytoplasmic microvilli [9,35,36,38,39] or by extending membrane protrusions into the vessel lumen [9,35,36,38,39,93]. The appearance of these micro-vascular changes corresponds to the duration of the ischemia and may be an adaptive EC response to altered hemodynamic conditions [9,93]. The functional significance of microvilli, microblebs and other morphological changes is not clear, but they may have a role in the production of delayed, post–ischemic hypoperfusion by increasing vascular resistance [9,93]**.** The extent of EC injury depends on the duration of ischemia and on the metabolic needs of the affected vascular system. The duration of experimental ischemia or acute anoxia required to cause damage varies for different organs. It takes approximately 10–15 minutes for irreversible damage to occur in brain [93,94,95]. After long–term ischemia and the following reperfusion, the decreased number of active capillary vessels is proportional to the ultrastructural lesions in ischemic vessels and underlying tissues and cells [9,35,36,38,39]**.** Cada and coworkers demonstrate that decreased CBF in aging rats produces deficits in visuospatial behavior after permanent surgical occlusion of both carotid arteries [96]. This deficit is coupled with metabolic abnormalities of the brain as visualized by quantitative COX histochemical mapping [96]. These results suggest that deficits in visuospatial learning are not exclusively the result of hippocampal dysfunction, but may be directly involved with altered oxidative energy metabolism in other integrative visuomotor regions identified in this study. They also suggest that chronic cerebrovascular ischemia in this aged rat model produces neurometabolic and behavioral alterations that may be relevant risk factors for the development of AD [96]. 

## 7. The Potential Role of Vasoactive Substances in the Endothelial Content during Ischemia/ Reperfusion

The synthesis and release of vasoactive substances, such as the endothelium–derived vasodilator NO and vasoconstrictor ET-1, regulate EC’s role in controlling vascular tone [65,97,98,99]. Aside from vascular tone NO regulates platelet aggregation, leukocyte adhesion, SMC proliferation, synaptic neurotransmission and cytotoxic/cytostatic actions of macrophages [84,99,100,101,102,103,106]. This labile molecule may carry out important biological roles both within the cell in which it is synthesized, and by interacting with nearby cells and molecules [107,108]**.** Three distinct isoforms of NOS derived from different genes generate NO: nNOS, iNOS, and endothelial NOS (eNOS) [97,98,99,100,101,102,103,104,105,106,107,108,109,110,111]**. **These isoforms are similar in structure and function [97,108,110,111]. eNOS was first purified and cloned from vascular endothelium, but is found in cardiac myocytes, blood platelets, brain cells [98,99,104,106,110,112] and in cellular compartments such as mitochondria [113,114]**.** The activity of eNOS is a major determinant of vascular tone and blood pressure. It is altered in diseases such as hypertension, diabetes, atherosclerosis, ischemia/reperfusion [41,87,104,105,115] and AD [73,89].

Excess NO is produced during excitotoxicity, inflammation and ischemia/reperfusion injury [116], and the oxidation products of NO, namely peroxynitrite and peroxynitrate. Also, ONOO**^–^** can generate the highly reactive hydroxyl radical, a more powerful oxidant than NO itself [17,102,116]. The increased nitrotyrosine immunoreactivity in AD is present in the neuronal cytoplasm of the cerebral cortex within regions of neurodegeneration, yet it is undetectable in corresponding control regions [17]. This distribution is essentially identical to that of free carbonyls [15]. The widespread occurrence of nitrotyrosine immunoreactivity in neurons [17] suggests that chronic oxidative damage is not restricted to long–lived polymers such as NFTs, but instead, reflects a generalized oxidative stress contributing to the pathogenesis of AD.

NOS positive neurons are present in subgroups throughout many regions of the brain [102]. Immunostaining for reduced NADPH-diaphorase, as well as nNOS and eNOS, reveals their presence in dendritic and axonal terminals that closely interact with the middle cerebral artery and cerebral microvessels [101,102,103,115]. The presence of L–arginine in astrocytes *in vivo* suggests that glia may store this chemical for NO production in brain** [101,115,117]. Moreover, glial cells exhibit an inflammatory response during infection or ischemic disease. They also release pro–inflammatory cytokines and synthesize and release NO** [117]. The large amount of NO that is released from glial cells via the expression of iNOS after their stimulation is neurotoxic, because it induces oxidative stress, mitochondrial dysfunction and excitotoxicity [101,106,118]. Hypoxic brain injury (acute or chronic) is associated with the formation of both NO [102,117,119,120,121,122] and the superoxide anion, which may react to form free radicals [17,106] and cause neurotoxicity** [101,102,103,106,119,120,123,124]. Further investigations into determining the exact ultrastructural localization of the different NOS isoforms in the brain vascular tree, neurons and glia in post–hypoxic and AD brain are warranted.

### 7.1. eNOS Involvement in the Cerebrovascular Tone

A dynamic balance of relaxing and constricting factors regulates cerebrovascular tone. Constitutively produced NO normally influences basal cerebral vascular tone, and mediates vascular responses to diverse stimuli [125] and cerebral vasodilation [117]**.** Vasorelaxation of brain microvessels is a feature of some diseases including chronic hypertension, diabetes, hypercholesterolemia, subarachnoid hemorrhage (SAH), and ischemia [41,117,125]. NO is also involved in regulating the cerebral circulation during hypercapnia [126,127] and focal [115,128-130] or global brain ischemia [127,131-135]. Furthermore, arginine–derived NO mediates the powerful effects of CO_2_ on cerebral circulation. NO synthesized by the action of nNOS participates in regulating basal CBF and is the major contributor to the hypercapnic CBF response [136]**. **Chronic inhibition of constitutive NO production increases EC permeability during various vascular diseases [8,87,95,98,99,110]. Due to its vascular effect, NO might improve tissue perfusion and exert a protective action. Overproduction, either by activation of nNOS by excitatory amino acids [137]**,** or by induction of iNOS in glial, vascular, or blood cells [132,133,134] during the ischemic episodes, might be deleterious. Mice with eNOS gene knockout exhibit a decrease in vascular relaxation. Thus, NO synthesized by eNOS protects against ischemic damage by increasing CBF, whereas NO produced by nNOS contributes to lesions [138,139]**.** The inhibition of NO synthesis by EC leads to increased intracellular oxidative stress, which induces neutrophils-EC interactions [9,37] and may promote the development and progression of vascular diseases such as atherosclerosis [41,105] and ischemia/reperfusion injury [6,9,35,117,125,140].

### 7.2. nNOS Expression and Regulation

Modification of nNOS expression in the entorhinal cortex and hippocampus occurs during AD [141]. Tissues containing the constitutive forms of NOS, like brain, kidney, and endothelium express dimethylargininase [142,143,144]. It regulates NO production by hydrolyzing free methylated arginine derivatives (effective endogenous inhibitors of NOS) [145]. The expression of dimethylargininase dramatically increases during AD [20]. Dimethylargininase abnormalities in the AD are the result of elevated levels of nitration from effective oxidants peroxynitrite or peroxynitrate [17,119,146]. However, the ultrastructural localization of dimethylargininase immunoreactivity in different cellular compartments of the AD brain or in Tg animal models of AD has yet to be described.

### 7.3. iNOS as a Mediator of Oxidation During AD

A variety of cells express iNOS in response to lipopolysaccharides, certain cytokines and ROS generators [8,97,99,101,102,103,104,112,115,117]**.** iNOS may be an important mediator of cytotoxicity in the brain because it produces much greater amounts of NO than either eNOS or nNOS [107]**. **Thorns and collaborators suggest that iNOS plays a role in the formation of NFT [141]. Iadecola and coworkers propose that iNOS contributes to ischemic brain damage [133]. The catalytic activities of iNOS enzymes or mRNA expression are evident in brain tissue after 2 hours of transient focal ischemia or 1–2 days after permanent focal ischemia [132,134]**.**

## 8. Subcellular Mechanisms Involved in the Development and Maturation of Human AD

**Figure 1:**
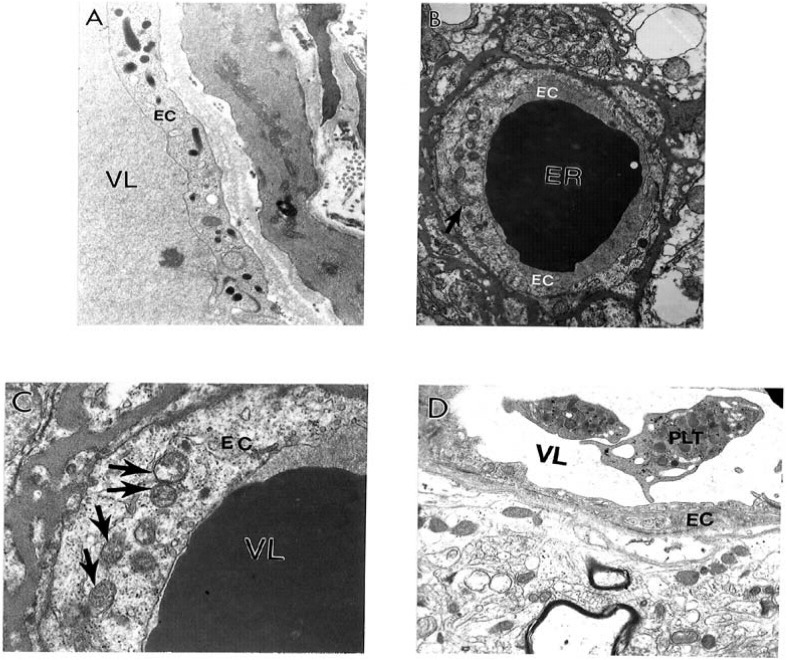
Ultrastructural features of the brain biopsy from the age-matched control (A) and AD (B–D) patients are characterized by heterogeneous morphology: A. An intact microvessel shows the absence of any particular abnormalities in the ultrastructure of endothelial cells (EC) and perivascular cells. Mitochondria in EC are intact. Original magnification: X 13,900. B. Short-post-mortem (<2 h) brain tissue from AD patients shows microvessels with severe damage such as the presence of clusters of mitochondria–derived lysosomes (single arrow) and necrotic changes in the ultrastructure of the EC and perivascular cells. Original magnification: X 8,300. C. Capillary endothelium (from Figure 1–B), under higher magnification, shows the presence of a cluster of damaged mitochondria (single arrows) containing positive mitochondrial DNA (mtDNA) signals visualized by using *in situ* hybridization following indirect colloidal gold decoration (17 nm gold particles). Original magnification: X 46,000. D. AD brain biopsy. EC occupied only the small part of the vessel wall. Perivascular cells show the presence of large mitochondria derived vacuoles in their matrix. Adhesion of the activated platelets (PLT) to damaged endothelium. Original magnification: 8,300. Abbreviations used in figures: EC–Endothelial cells; ER–Erythrocyte; PLT–Platelets; VL–Vessel lumen (reprinted from [172] with permission).

Ultrastructural features of the brain biopsy from the age-matched control (Figure 1A) and AD (Figure 1B–D) patients are characterized by heterogeneous morphology. The EC in intact microvessels did not show visible changes. Mitochondria in EC are intact (Figure 1A). 

**Figure 2:**
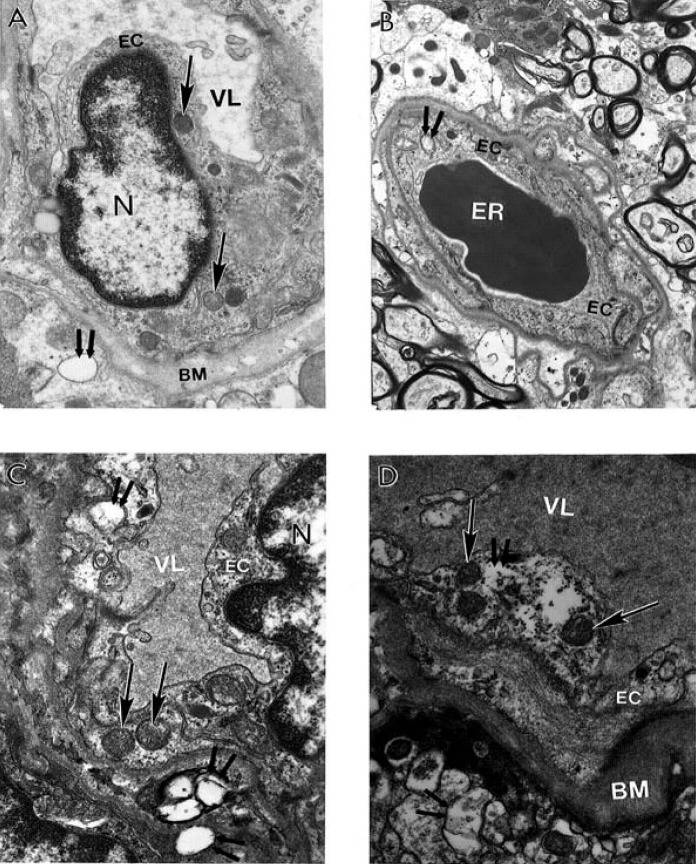
Ultrastructural features of brain microvessels from AD brains characterized by heterogeneous morphology. mitochondria abnormality appeared A. Undamaged microvessel endothelium did not show any particular changes in their ultrastructure. Mitochondria also were intact (single arrows). However, the perivascular spaces contained large vacuolar structures (double arrow). Original magnification X 13,000. B. Vascular EC shows the presence of degenerative mitochondria (double arrow). Original magnification X 6,600. C. The presence of electron-dense hypoxic mitochondria (single arrows) coexists with the formation of mitochondria derived lysosomal structure in the cytoplasmic matrix of EC and perivascular cells (indicated by double arrow). Original magnification X 20,000. D. To be a permanent feature of vascular endothelium and perivascular cells where damage became visible (single and double arrows indicate hypoxic and completely damaged mitochondria, respectively). Original magnification X 20,000. Abbreviations used in figures: BM–basal membrane of endothelium; EC–endothelial cell; ER–erythrocyte; VL–vessel lumen (reprinted from [87] with permission).

Contrary to this observation, short–post–mortem (<2 h) brain tissues, from AD patients, show microvessels with severe damage, which characterizes the presence of clusters of mitochondria derived lysosomes and necrotic changes in their ultrastructure (Figure 1B). The capillary endothelium shows the presence of a cluster of damaged mitochondria containing positive mitochondrial DNA (mtDNA) signals visualized by using *in situ* hybridization following indirect 17 nm colloidal gold decoration (Figure 1C). In addition, in AD brain microvessels EC occupied only the small part of the vessel wall. Perivascular cells show the presence of large mitochondria derived vacuoles in their matrix. Adhesion of the activated platelets (PLT) to damaged endothelium appeared to be hallmark of these microvessels (Figure 1D). However, undamaged microvessel endothelium did not show any particular changes in their ultrastructure. Mitochondria also were intact (Figure 2A). However, the perivascular spaces contained large vacuolar structures (see Figure 2A). At the same time often the microvessel EC shows the presence of degenerative mitochondria (Figure 2B). The presence of electron-dense hypoxic mitochondria coexists with the formation of mitochondria derived lysosomal structure in the cytoplasmic matrix of EC and perivascular cells (Figure 2C). The mitochondria abnormality appeared to be a permanent feature of vascular endothelium and perivascular cells where damage became visible which characterizes the presence of a hypoxic and completely damaged mitochondria (Figure 2D). 

**Figure 3:**
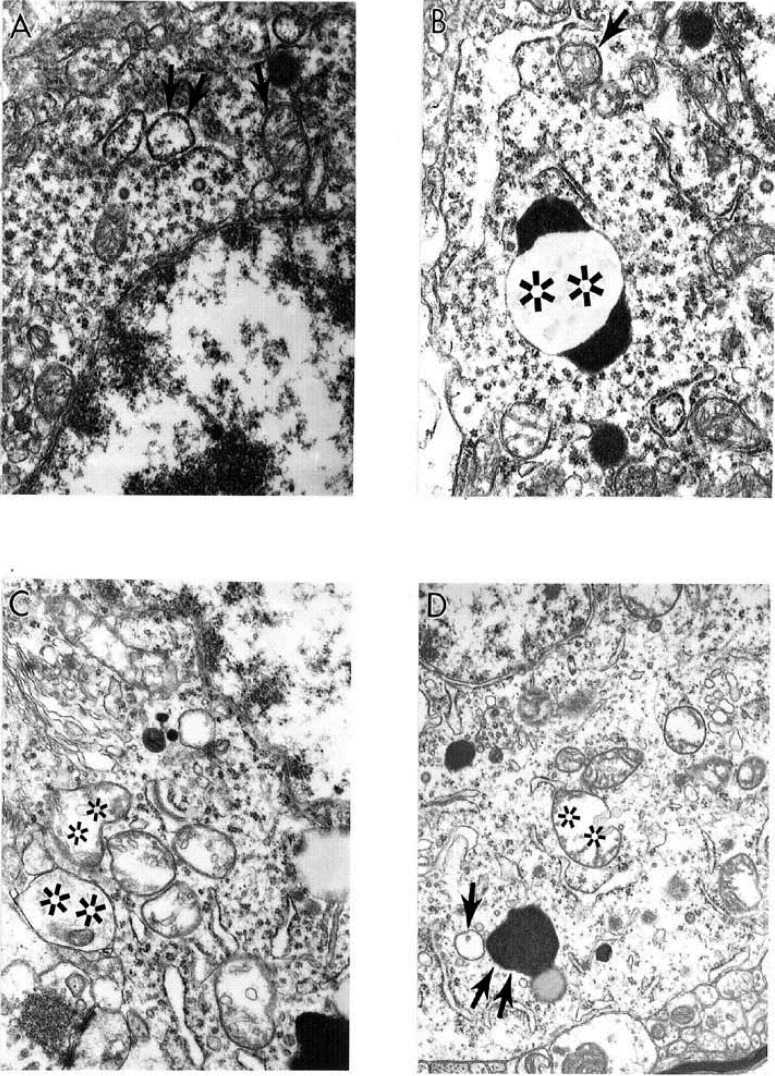
The ultrastructural characteristics of neuronal mitochondria damage from AD brain biopsy. Neurons with different degree of ultrastructural lesions. In the neuronal cell body partially and completely damaged mitochondria (indicated by single arrows and double asterisk respectively in A and C). The lesioned mitochondria appeared to be a major substrate for the lipofuscin formation (double arrow). The electron dense hypoxic mitochondria are seen throughout the cell body and characterize the abnormal mitochondrial cristae. Original magnification: A and B X20,000 respectively. C and D X 16,000 respectively (reprinted from [172] with permission).

We demonstrate in recent work that cortical neurons from AD brain biopsies have selective localization of mitochondria abnormalities in the cell body [1,3,53,59,87,89]. The majority of the neurons, which closely associate with the lesioned vessels, possess differing degrees of ultrastructural abnormality. The ultrastructural characteristics of neuronal mitochondria damage from AD brain biopsies show the presence of neurons with differing degrees of ultrastructural lesions (Figure 3). In the neuronal cell body partially and completely damaged mitochondria appeared to be hallmark of these neurons (Figure 3A and Figure 3C). The lesioned mitochondria appeared to be a major substrate for the lipofuscin formation. The electron dense hypoxic mitochondria are seen throughout the cell body and characterize the abnormal mitochondrial cristae (in Figure 3B and Figure 3D).

**Figure 4:**
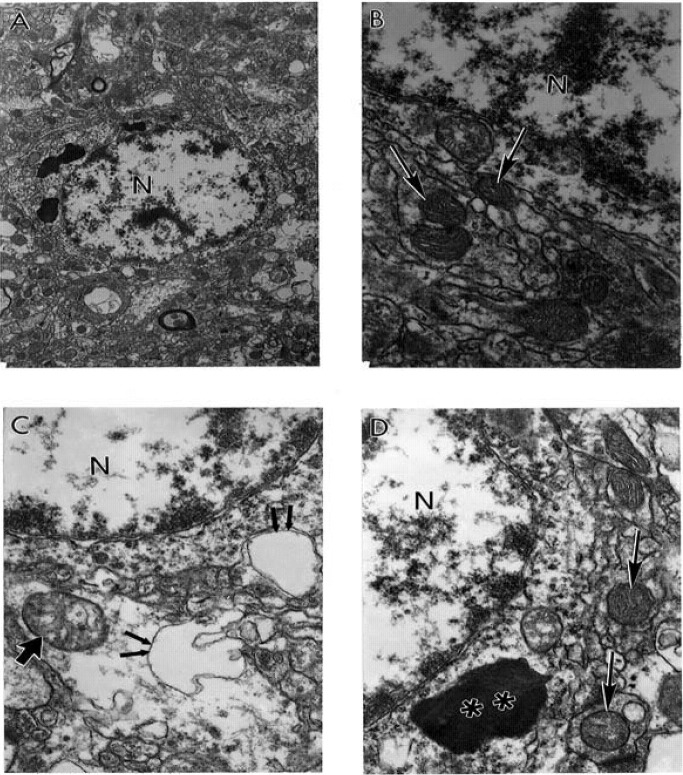
The ultrastructural characteristics of the neuronal mitochondria from AD brain biopsy. A. Neurons with different degrees of ultrastructural lesions. Partially and completely damaged mitochondria are mostly located in the neuronal cell body and coexist with lipofuscin formation. Original magnification X 5,000. B. Large numbers of electron-dense hypoxic mitochondria (indicated by single arrows) were present throughout the cell body and characterized the abnormal mitochondrial cristae. Original magnification: X 20,000. C. Partially (indicated by single arrow) and completely damaged (double arrow) mitochondria. Original magnification X 20,000. D. The neuronal cell body shows the presence of hypoxic mitochondria (indicated by single arrows) close to lipofuscin (double asterisk). Original magnification X 20,000. Abbreviations used in figure: N– neuronal nucleus (reprinted from [87] with permission).

Neurons with different degrees of ultrastructural lesions were seen throughout cortex (Figure 4A). Partially and completely damaged mitochondria are mostly located in the neuronal cell body and coexist with lipofuscin formation (Figure 4A). Moreover, another feature of these neurons appears to be the presence of large numbers of electron-dense hypoxic mitochondria, which were present throughout the cell body and characterized the abnormal mitochondrial cristae (Figure 4B). In addition, these abnormalities coexist with the presence of clusters of the partially and completely damaged mitochondria (Figure 4C). The neuronal cell body always shows the presence of hypoxic mitochondria close to lipofuscin (Figure 4D).

**Figure 5:**
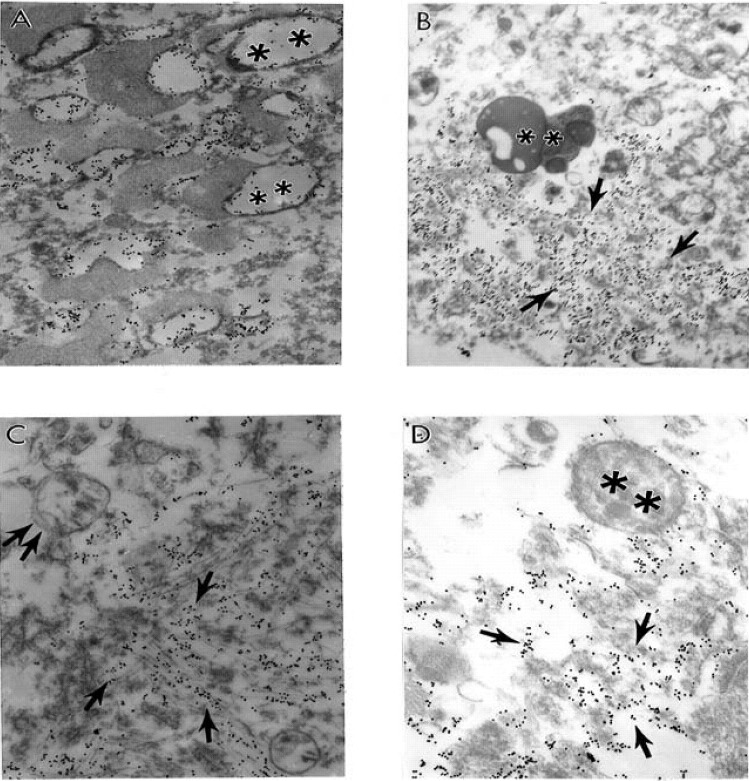
The features of wild type (A) mitochondrial DNA (mtDNA) and 8-OHG (B–D) staining in the hippocampus of short post–mortem (<2 h) human AD brain. Post–mortem AD hippocampus shows that wild type mtDNA (17 nm gold) is associated with severely damaged mitochondria and mitochondria derived lysosomes (double asterisk). Any area containing lipofuscin did not show mtDNA containing positive gold particles. Original magnification X 26,000. B–D: Features of 8-OHG staining in post–mortem AD brain. 8-OHG containing positive signals (17 nm gold particles) was seen throughout neuronal cell body and within in the matrix of damaged mitochondria (single arrow). Non–damaged mitochondria (indicated by double arrows in Figure.5C) and lipofuscin (double asterisk in Figure. B and D) do not contain 8-OHG positive gold particles. Original magnification: X16,000, 26,000 and 33,000 respectively B, C and D (reprinted from [172] with permission).

The features of wild type mitochondrial DNA (mtDNA) and 8-OHG staining in the hippocampus of short post–mortem (<2 h) human AD brain shows that wild type mtDNA (17 nm gold) is associated with severely damaged mitochondria and mitochondria derived lysosomes (Figure 5A). However, the area containing lipofuscin did not show any mtDNA containing positive gold particles (Figure 5A). Features of 8-OHG staining in post–mortem AD brain shows that the 8-OHG containing positive signals (17 nm gold particles) was seen throughout neuronal cell body and within in the matrix of damaged mitochondria (Figure 5B–D). However, non–damaged mitochondria (in Figure 5C) and lipofuscin (in Figure 5B and Figure 5D) do not contain 8-OHG positive gold particles.

**Figure 6:**
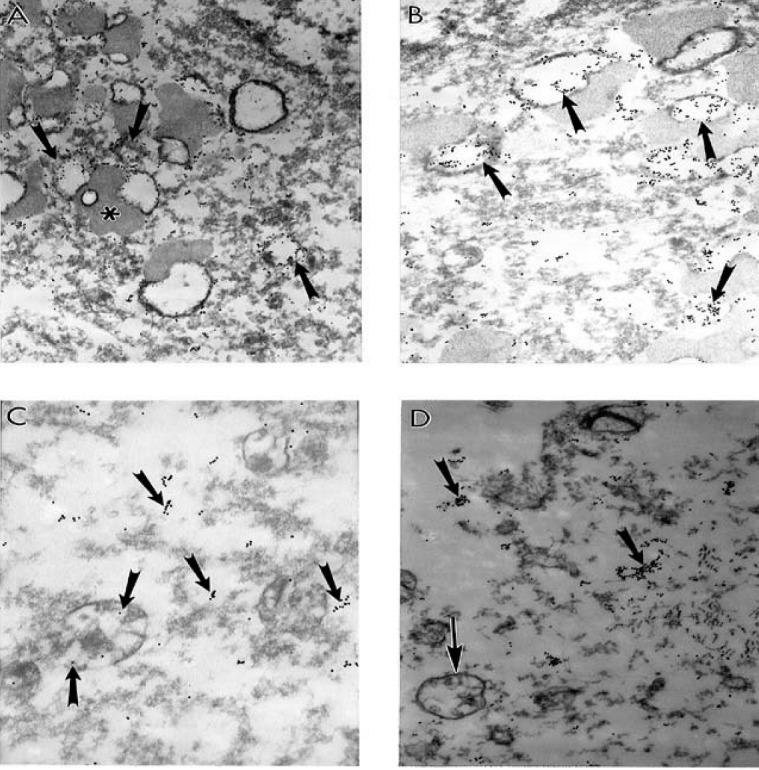
The features of wild, 5kb deleted mitochondria DNA (mtDNA), and COX immunoreactivity in the hippocampus of a postmortem human AD case. A and B. Hippocampal neuron shows wild type mtDNA containing positive signals (17 nm colloidal gold) detection were seen in the completely damaged mitochondria or mitochondria derived lysosomes (single arrows). Areas containing lipofuscin (asterisk) did not show any mtDNA containing positive signals. Magnification X 26,000 and X 20,000, respectively A and B. C. 5kb deleted mtDNA containing gold particles (17 nm) were mostly located in mitochondria–derived lysosomes (single arrows). Original magnification X 33,000. D. Damaged, abnormal mitochondria shows COX positive containing gold particles in the matrix (single arrows, colloidal gold 17 nm). Original magnification X 26,000 (reprinted from [172] with permission).

The features of wild type (in Figure. 6A–B), 5kb deleted mtDNA (in Figure 6C), and COX immunoreactivity (in Figure. 6D) in the hippocampus of a postmortem human AD case shows wild type mtDNA containing positive signals (17 nm colloidal gold) detection were seen in the completely damaged mitochondria or mitochondria derived lysosomes. Any areas containing lipofuscin did not show mtDNA–containing positive signals (in Figure 6A–B). In addition, the 5kb deleted mtDNA containing gold particles (17 nm) were mostly located in mitochondria–derived lysosomes (in Figure 6C). Damaged, abnormal mitochondria shows COX positive containing gold particles in their matrix (in Figure 6D). 

Mitochondrial lesions and lipofuscinogenesis are also present in other cellular compartments of the brain parenchyma. Glial cells at the damaged area, also characterized by the accumulation of lipofuscin and mitochondria–derived lysosomes appear to be a major component and source for these substrates (data not shown). In addition, glial cells also show the intracellular accumulation of different sized amyloid deposits, and they are accompanied by the presence of giant–sized lipid–laden vacuoles and mitochondria–derived lysosomes**. **

Quantitative morphometric measurements of the percentage of the different types of mitochondria (normal, partially damaged and completely damaged) indicate that age-matched control groups have a significantly higher percentage of normal mitochondria, compared to completely damaged mitochondria from AD cases [2,3,51,56]. No significant differences between partially damaged mitochondria are seen in both groups, indicating that aging induces damage to mitochondria. However, the main differences between the age-matched controls and AD cases appear to be significant differences in the percentage of the normal and completely damaged mitochondria [2,3,51,56].

## 9. Antioxidant Application for the Treatment of AD

AD treatment has yet to yield a successful therapy that addresses the cause of injury found in AD brain biopsies [11]. Of the various theories proposed for AD etiology, ROS generation is cited as a common factor based on several cellular, molecular, and animal model studies of AD. During aging, ROS may play a large role in cell death, an important factor responsible for disease progression [66]. Efforts to reduce the pathology associated with ROS via antioxidants seem to offer new hope to patients suffering from this devastating disease [11].

Mitochondria has been considered a primary target in the search for age-related cognitively impaired conditions to restore cognitive function including treatments for dementia [11,147,148,149,150,151,152,153,154]. This is because the brain, which is characterized by a high energy metabolism and abundance of oxidizable materials such as polyunsaturated fatty acids and neuropeptides, is exceedingly susceptible to oxidative damage [155], which is known to cause mitochondrial dysfunction [154]. Furthermore, Aβ is reported to promote an excess accumulation of intracellular Ca^2+^ into mitochondria, inducing mitochondrial permeability pores to open, damaging mitochondrial structures. which in return increase the production of defective mitochondria, decrease mitochondrial trafficking, and alter mitochondrial dynamics in neurons affected by AD [156]. The association of mitochondrial activity with the antioxidant capacity of certain micronutrients such as alpha–Lipoic acid (LA), a coenzyme essential for the maintenance of energy homeostasis in mitochondria, has been shown to influence cognitive function in an different animal species [147,148,149,150,151,152,153,154].

Previous studies have demonstrated the potential protective effects of selective mitochondrial antioxidant treatments on brain mitochondria from aged rats [147,148,149,150,152,157]. When treating aged rats with selective mitochondrial antioxidants, such as ALCAR and LA, they were able to reduce OS and restore cognitive function and mitochondrial structural abnormalities in all parenchymal cells [147,151,157,158]. It is important to note that the oxidative damage is associated with mitochondria early in AD progression [159]. In addition, to study mitochondrial decay and oxidative damage resulting from aging, an examination into the activities and kinetics of the mitochondrial complexes (a hallmark of the mitochondrial ability to produce energy) was performed, and showed that mitochondrial complexes can be restored by selective mitochondrial antioxidant treatment [158]. This established that in the brain mitochondria of old rats, when compared with that of young rats, there were significantly decreased endogenous antioxidants and less superoxide dismutase activity; more oxidative damage to lipids and proteins; and decreased activities of the mitochondrial complex I, IV and V [158]. In regards to this, mutant proteins associated with AD are reported to block the transport of nuclear–encoded mitochondrial proteins to mitochondria, interact with mitochondrial proteins and disrupt the electron transport chain, induce free radicals, cause mitochondrial dysfunction, and, ultimately, damage neurons [160]. Moreover, the mitochondrial complex I showed a decrease in binding affinity (increase in K(m)) for substrates. Feeding LA/ALCAR to old rats partially restored age-associated mitochondrial dysfunction compared to young rats. These results indicate that oxidative mitochondrial decay plays an important role in brain aging, inducing the generation of free radicals that leads to oxidative damage in postmortem brain neurons from AD patients and in brain neurons from cell models and transgenic mouse models of AD [161], and that a combination of nutrients targeting mitochondria, such as LA/ALCAR, could ameliorate mitochondrial decay through preventing mitochondrial oxidative damage [158]. In a recent study [147] we were able to demonstrate that the integrity of mitochondrial ultrastructure, which is dependent on aging, could be improvement in old rat brain mitochondria when compared to the control group by using antioxidant treatments [147]. In contrast, neurons obtained from aged control groups showed a series of mitochondrial abnormalities, such as the presence of giant mitochondria and mitochondria with partial or complete damaged cristae. Targeting mitochondrial OS improved the overall cognitive ability of aged rats [148,149,150,152,157,162] and aged dogs [153].

Reid and colleagues [163] noted that there is epidemiological evidence that links vascular diseases, such as hypercholesterolemia, with an increased incidence of AD. While no theory has yielded a satisfactory explanation for the pathological changes that lead to neurodegeneration and cognitive dysfunction [163], vascular risk factors seem to offer the most interesting results [2,164]. The relationship between hypercholesterolemia and AD arose in great extent from ApoE4, a known risk factor for AD and a major carrier of cholesterol in the CNS. The detrimental processes of ApoE4 have been shown to influence AD pathological processes, including lipid homeostasis and NFT formation [165], which suggests that brain vascular alternations play a key role in the progression of AD [163]. ApoE4 mechanisms that contribute to the neurodegeneration of the brain could offer strong insights into AD susceptibility. For example, it was shown in a rat model that ApoE levels would increase as a response to peripheral nerve injury [165], implicating the role of ApoE as a repair mechanism. If the delivery of lipophilic antioxidants is impaired due to ApoE4, this could lead to OS [166]. It has been proven that AL can improve memory deficits in animal models of AD and reduce cognitive deficit in AD patients [155]. The contribution of fatty acids in these cerebrovascular processes, and their effect on AD pathogenesis is still uncertain, but the suggestion that they can elicit neuronal overexcitation and synaptic depression as contributor factors to AD is suggested [167].

Several therapeutic strategies have been developed to treat AD, including anti–inflammatory, anti–oxidant, and herbal treatment approaches. These have been tested in animal and cellular models of AD and in clinical trials with AD subjects. In AD animal models and cell models, herbal extracts appear to have fewer adverse effects than beneficial effects on cognitive functions because of their antioxidant, anti-inflammatory properties [168]. We analyzed the effect of mitochondrial antioxidants ALCAR and LA as a treatment model for AD on ApoE4 transgenic mice [154]. The decrease in cerebrovascular oxygen levels seen in AD patients led to the hypothesis that hypoperfusion in the CBF, which over time causes OS and mitochondrial damage, was the main cause of ApoE–related cognitive deficits was seen in AD patients with ApoE4 overexpression [169,170,171]. Our study demonstrated for the first time, that ApoE4 caused brain hypoperfusion by gradually reducing CBF when compared to a control group. Structural damage of vascular wall cells, especially in mitochondria, seems to play a key role in the generation of ROS, resulting in oxidative damage to the neuron and inducing pathological factors associated with AD [154]. Therefore, we believe that expanding the focus of study in AD towards mitochondrial pathobiology as a treatment strategy will be able to open new and more successful effective treatment strategies for this devastating disease [62,67,147,154,164,172].

## 10. Conclusions 

In this review we indicate that chronic vascular hypoperfusion is a part of the common underlying mechanisms involved in the initiation and development of neurodegenerative disorders such as stroke and arteriosclerosis. In this regard, it appears that the central initiating factor for vascular abnormality is mitochondrial damage and a sum of elucidators for the imbalance in the activity of NOS isoforms, ET-1, oxidative stress markers, mtDNA and mitochondrial enzymes in the vascular wall and in brain parenchymal cells. This is believed to be due to their predominance in CVA and AD. We hypothesize that an imbalance between the NOS species and the endothelium, along with antioxidant system deficiencies, are predominant brain features of stroke and AD patients. Elevated chronic hypoperfusion and physical distortion of tissue are likely to contribute to the collapse of post–ischemic/hypoxic or AD vessels. We theorize that future eliminating mitochondrial abnormalities can be considered as a new and more effective treatment strategies for this devastative disease in the near future. 
